# Rhubarb polysaccharide and berberine co-assembled nanoparticles ameliorate ulcerative colitis by regulating the intestinal flora

**DOI:** 10.3389/fphar.2023.1184183

**Published:** 2023-06-20

**Authors:** Yifan Feng, Chenyang Wu, Huan Chen, Tingting Zheng, Hanyi Ye, Jinrui Wang, Yinghua Zhang, Jia Gao, Ying Li, Zhengqi Dong

**Affiliations:** ^1^ Drug Delivery Research Center, Institute of Medicinal Plant Development, Chines Academy of Medical Sciences, Peking Union Medical College, Beijing, China; ^2^ Jilin Provincial Academy of Chinese Medicine, Changchun, China; ^3^ Key Laboratory of Bioactive Substances and Resources Utilization of Chinese Herbal Medicine, Ministry of Education, Chinese Academy of Medical Sciences, Peking Union Medical College, Beijing, China; ^4^ Key Laboratory of New Drug Discovery Based on Classic Chinese Medicine Prescription, Beijing, China; ^5^ Beijing Key Laboratory of Innovative Drug Discovery of Traditional Chinese Medicine (Natural Medicine) and Translational Medicine, Beijing, China

**Keywords:** rhubarb polysaccharide, berberine, co-assembly, nanodrug, gut microbiota, ulcerative colitis

## Abstract

**Introduction:** Inflammatory bowel disease (IBD) affects about 7 million people globally, which is a chronic inflammatory condition of the gastrointestinal tract caused by gut microbiota alterations, immune dysregulation, genetic and environmental factors. Nanoparticles (NPs) deliver an active natural compound to a site harbored by disordered microbiota, they are used to interact, target and act intentionally on microbiota. Although there is accumulating evidence indicating that berberine and polysaccharide play an important role in IBD via regulating microbiota, there is limited research that presents a complete picture of exactly how their carrier-free co-assembled nanodrug affects IBD.

**Methods:** The study establishes the carrier-free NPs formed by berberine and rhubarb polysaccharide based on the combination theory of *Rheum palmatum L.* and *Coptis chinensis Franch.*, and characterizes the NPs. The IBD treatment efficacy of NPs are evaluated via IBD efficacy index, and explore the mechanism of NPs via 16S rRNA test and immunohistochemistry including occludin and zonula occludens-1.

**Results:** The results showed that DHP and BBR were co-assembled to nanoparticles, and the BD can effectively relieve the symptoms of UC mouse induced by DSS via regulating gut microbiota and repair the gut barrier integrity, because BD have a longer retention on the colon tissue and react with the microbiota and mucus thoroughly. Interestingly, BD can enrich more probiotic than free BBR and DHP.

**Discussion:** This design provides a better strategy and encourages future studies on IBD treatment via regulating gut microbiota and the design of novel plant polysaccharide based carrier-free co-assembly therapies.

## 1 Introduction

Inflammatory bowel disease (IBD) is a chronic and life-threatening inflammatory disease of the gastrointestinal tissue, characterized by episodes of intestinal inflammation. The main types of IBD are ulcerative colitis (UC) and Crohn’s disease (CD), resulting from the interaction of genetic and environmental factors (Seyedian et al., 2019). With the improvement of living standards and changes in lifestyle habits, the number of patients suffering from IBD in the world is increasing year by year. In particular, UC, one of the two main branches of IBD severely reduces the quality of survival and has a high risk of recurrence. Although the mechanisms involved are still being explored, it is certain that the alteration of the gut microbiota can play a protective or damaging role. The intestinal flora has become a potential target for the treatment of diseases (Fan and Pedersen, 2021).

Berberine (BBR) is an alkaloid that can be extracted from *Coptis chinensis* Franch*.*, and it has an amino structure (Habtemariam, 2020). There is increasing evidence that BBR can improve UC, regulate the intestinal microflora, and keep the balance of the T regulatory (Treg) and T helper 17 (Th17) cell by improving metabolic disorders (Cui et al., 2018). It reduces the expression of oncostatin M (OSM) and the ligand (OSMR), thereby affecting the JAK–STAT pathway mediated by it and inhibiting the over-activation of human intestinal stromal cells (Li et al., 2020). BBR can decrease the food intake, body weight, blood sugar, and HbA1c levels of mice, which can effectively restore the content of short-chain fatty acids, reduce serum LPS levels, relieve intestinal inflammation, and repair the intestinal barrier structure (Zhang et al., 2019). BBR intervention changes the microbial composition of mice and increases the relative abundance of short-chain fatty acid-producing bacteria, such as *Butyricimonas*, *Coprococcus*, and *Ruminococcus*. When reducing the number of opportunistic pathogens such as *Prevotella* and *Proteus*, the abundance of other probiotics such as *Lactobacillus* and *Akkermansia* genera also increased, so BBR can relieve intestinal inflammation by modulating the composition of the intestinal flora.

In recent years, studies have gradually shown that some polysaccharide excipients, such as chitosan, pectin, and alginate, can also interact with the intestinal flora. Gut microbiota regulation can achieve specific effects, such as alleviating colitis symptoms and lowering hyperlipidemia. Therefore, as a key part of constructing a drug delivery system, the potential use of excipients based on interactions with the gut microbiota must be considered. The complex and diverse chemical components of Chinese medicine, including polysaccharides, glycosides, organic acids, alkaloids, steroids, triterpenes, and proteins, are prone to molecular recognition and a co-assembly of chemical components after decoction and dissolution, forming co-assembled nanoparticles (Zhao et al., 2020). The formation mechanism of nanoparticles in Chinese medicine is generally believed to be induced by non-covalent bonding, such as hydrogen bonding, van der Waals forces, π–π stacking, fractional complexation, and electrostatic interactions (Duan et al., 2015; Huang et al., 2015; Ma et al., 2016). With the in-depth verification of the properties and functions of polysaccharide components, polysaccharide-based NPs were proved to improve the solubility of insoluble drugs, enhance the intestinal mucin targeting ability, and realize a controlled and sustained release of drugs (Da Silva et al., 2014). *Rheum palmatum* L*.*, a kind of traditional Chinese medicine, is a classic compatibility drug with *Coptis chinensis* Franch*.* The rhubarb polysaccharide (DHP) is mainly derived from *Rheum palmatum* L*.*, as a natural polysaccharide; it has the advantages of biodegradability, low immunogenicity, and low toxicity. The structure of the DHP contains a large number of groups such as hydroxyl groups, which are prerequisites for the formation of co-assembly systems with BBR through electrostatic and hydrogen bonding interactions.

Co-assembled nanoparticles composed of pharmacological molecules have been proved to strengthen the intestinal barrier, regulate the gut microbiota, and realize the nano-immunotherapy of IBD. The nature or type of nanoparticles, their structures, their concentrations, and the interacted bacteria studied may all make a difference in their therapeutic effect. Some natural drugs were found to readily form hydrogen bonds between intestinal mucin and nanoparticles, exhibiting specific mucin-binding functions. Some strain-specific bacterial adhesins were also found to selectively attach to glycoarrays presented by mucin. This makes them a potentially new natural drug delivery nanocarrier that could replace synthetic nanomaterials.

**FIGURE 1:**
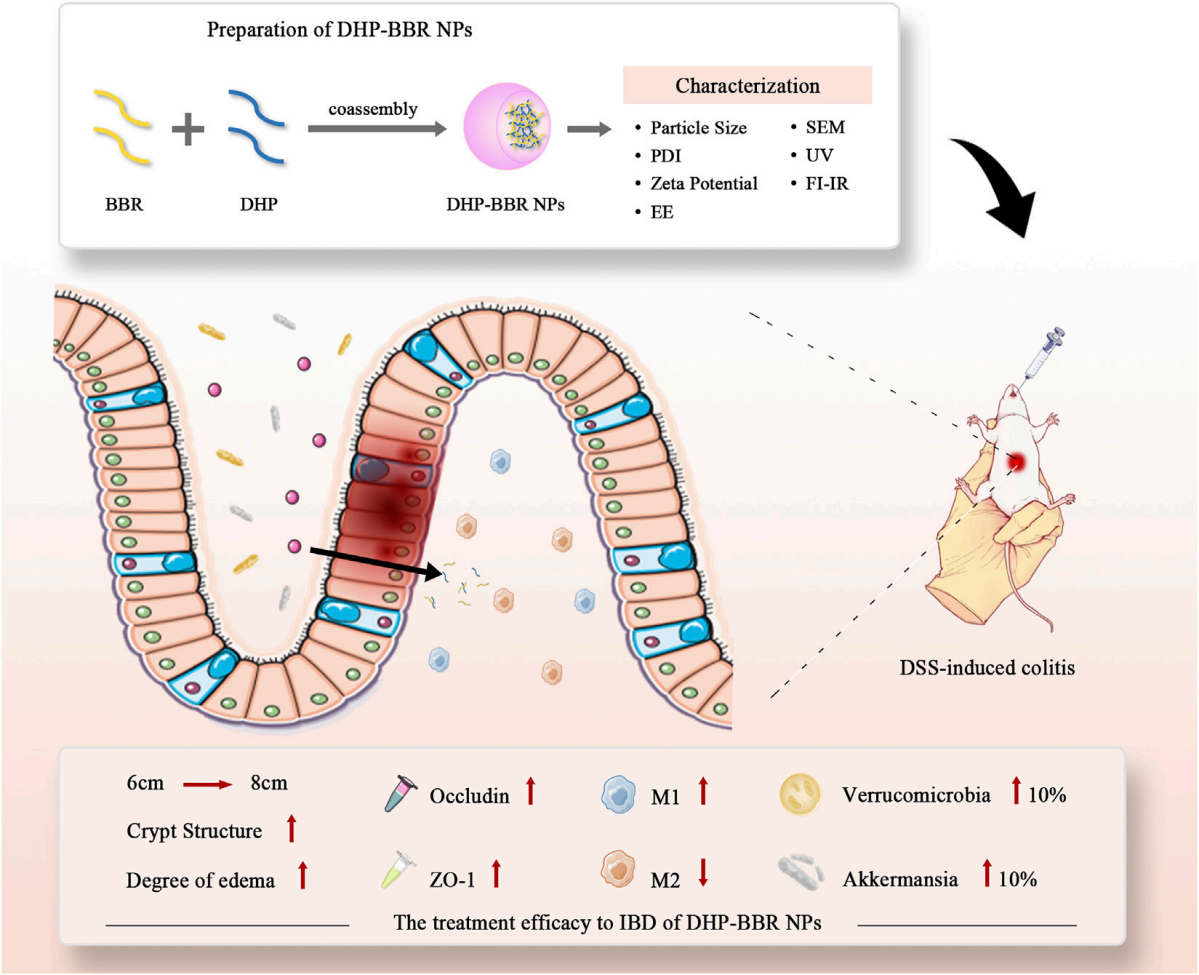
Scheme of co-assembly-nanoparticle construction and its effect on IBD and gut microbiota regulation.

Therefore, based on the co-assembly strategy between natural chemical substances, we have carried out in-depth research on the follow-up preparations related to the DHP and BBR and explored the self-assembly of two kinds of compounds to form a nano-formulation. The possibility of gastrointestinal protection and alleviation of UC were studied. Gut microbiota regulation, immunity regulation, and gastrointestinal barrier integrity repair were studied to explore the mechanism of the co-assembly of BBR–DHP NPs (BD). The process of preparing nanoparticles and their efficacy evaluation is shown here (Figure 1). We found that the DHP and BBR can co-assemble to form nanoparticles with a good size dispersion. The nanoparticles effectively ameliorate UC in mice. The possible mechanisms involved in regulating inflammatory factors are the tight junction protein and gut microbiota. The design provides a reference for the fabrication of natural polysaccharide-based NPs as a drug delivery system for UC treatment.

## 2 Materials and methods

### 2.1 Materials

The DHP was purchased from Shanghai Ronghe Pharmaceutical Co., Ltd. BBR was obtained from Nanjing Zelang Co., Ltd. Pepsin and trypsin were purchased from Shanghai MacLean Biochemical Technology Co., Ltd. Occludin and zonula occludens-1 (ZO-1) antibodies were purchased from Abcam, USA. Sodium dextran sulfate was purchased from MP, USA. A volume of 4% of a tissue cell fixative was purchased from Beijing Solaibao Biotechnology Co., Ltd. 1,1′-Dioctadecyl-3,3,3′,3′-tetramethylindocarbocyanine perchlorate (DIL) was purchased from Beyotime Biotechnology Co., Ltd. The fluorescent blocker containing DAPI was purchased from Beijing ZSGB-Biotechnology Co., Ltd.

### 2.2 Characterization of the DHP

The FT-IR spectra of the DHP were recorded in the range of 4,000–400 cm^-1^ using a Nicolet IS10 FT-IR spectrometer (Nicolet iS10, Wisconsin, USA). The release media measured the content of the DHP using a high-performance liquid chromatograph (Prominence LC-20AD, Kyoto, Japan). The mobile phase was A (17% acetonitrile), and B was 83% potassium dihydrogen phosphate (0.05 M; pH = 6.70) in H_2_O, gradient elution was 20%–45% A in 0–5 min and 45% of A in 5–7 min, the liquid velocity was 1 mL min^-1^, and the injection volume was 20 μL. ^1^H-NMR (600 MHz) spectra of the DHP were tested by employing a Bruker Avance Ⅲ 600 MHz NMR instrument (Bruker Avance Ⅲ 600 MHz, Salbruken, Germany). D_2_O was used as a solvent and internal standard (4.70 ppm) of the DHP. The thermal characteristics of the DHP is assessed using a differential scanning calorimeter (Nechi NETZSCH STA 449 F3/F5, Selb, Germany) under a dry nitrogen atmosphere and black aluminum pot as control. The instrument parameter was set at 10°C·min,^-1^ and the scanning range was 10°C–300°C.

### 2.3 Preparation of BD

We weighed an appropriate amount of the DHP to prepare a 1.0 mg mL^-1^ aqueous solution of the DHP and weighed an appropriate amount of BBR to prepare a 2.0 mg mL^-1^ BBR ethanol solution. Nanoparticles were prepared according to the volume ratio of the DHP to BBR of 1:1 and their mass ratio of 1:2. The ethanol solution of BBR was added dropwise to the aqueous solution of the DHP with a syringe (0.4 × 13 mm) at 600 rpm. The stirring condition at room temperature was continued for 2 h, followed by sonication for 30 min to form a nano-suspension. After freeze-drying, the freeze-dried powder was reconstituted in water and dialyzed in a dialysis bag with a molecular weight cut-off of 2,500 Da for about 12 h, and the freeze-dried yellow powder is the pure nanoparticles of DHP–BBR.

### 2.4 Characterization of BD

We took 0.8 mL of the DHP–BBR nano-suspension and placed it in a potential cell and measured its particle size, polydispersity index (PDI), and zeta potential with a dynamic light scattering instrument. We also took an appropriate amount of lyophilized non-pure BD, re-dissolved it in water, and carried out its dialysis. An appropriate amount of pure BD after dialysis was lyophilized and reconstituted in water, for a comparison of the particle size, PDI, and zeta potential before and after dialysis; we investigated whether freeze-drying affects the properties of BD.

We took 0.5 mL of the BD solution and carried out ultrafiltration centrifugation with an ultrafiltration centrifuge tube with a molecular weight cut-off of 1,500 Da and centrifugation conditions of 3,000 rpm for 20 min. The newly prepared nanoparticles were freeze-dried, and fresh nanoparticles were taken and coated with gold for 6 min and then observed and photographed by a scanning electron microscope at different magnifications.

The properties of the functional groups on the surfaces of the DHP, BBR, and BD were analyzed by UV–Vis spectroscopy and FT-IR spectroscopy, scanning in the wavelength range of 200–800 nm. To measure the storage stability of nanoparticles, we took an appropriate amount of lyophilized BD, reconstituted it, and placed it in a potentiometric cell to measure its particle size, PDI, and zeta potential with a dynamic light scattering instrument for 1 week.

### 2.5 Stability study of BD

We investigated the stability of BD in simulated gastric and intestinal fluids. First, we prepared the simulated gastric fluid. A volume of 16.4 mL of 1 mol/L dilute hydrochloric acid was added to 800 mL of distilled water; 10 g of pepsin was weighed, mixed, and diluted to 1 L with distilled water, and the simulated gastric fluid is obtained by filtering through the aqueous phase. Next, we prepared simulated intestinal fluids. A volume of 6.8 g of potassium dihydrogen phosphate was dissolved in 500 mL of distilled water; we adjusted the pH to 6.8 with 0.1 mol L^-1^ of NaOH, and 10 g of trypsin was dissolved in deionized water (DI). After mixing, the solution was diluted to 1 L with distilled water. The simulated intestinal fluid is obtained by filtering through the aqueous phase. BD was incubated with the artificial gastrointestinal fluid at 1:4 (v/v) in an air bath at 37°C, and 1 mL of it was taken at 0, 1, 2, 4, 6, 8, and 12 h to determine their respective particle sizes. Each sample was measured three times.

### 2.6 *In vitro* release experiments

The *in vitro* dialysis bag diffusion method was used to simulate the *in vivo* release process of nanoparticles. An appropriate amount of BD was precisely weighed and prepared into an aqueous solution of 1.0 mg·mL^-1^ with a volume of 2 mL. For activation treatment, the prepared BD solution was added to the dialysis bag, and 30 mL of PBS (pH = 7.4) solution was used as the release medium. The aforementioned process was carried out in an air bath at 37°C with a rotation speed of 100 r·min-1. At specific time points (0, 1, 2, 4, 6, 8, 10, and 12 h), 500 μL of the release medium was taken out, the content of BBR in the release medium was determined by HPLC, and the same volume of the release medium was supplemented at the same time. Then, we calculated the cumulative release rate and drew a release curve.

### 2.7 Evaluation of anti-ulcerative colitis efficacy *in vivo*

#### 2.7.1 Establishment of the mice model

The UC model was established by the intervention of a dextran sodium sulfate (DSS) aqueous solution. First, the model establishment method was explored. It is a gradient intervention method; that is, 5% DSS aqueous solution intervenes continuously for 2 days, and 3% DSS aqueous solution intervenes continuously for 3 days. The survival of the mice was observed, the body weight of the mice was recorded daily, and the scores were recorded in terms of fecal consistency and bloody stools. Finally, the Disease Activity Index (DAI) score was calculated as the sum of the weight change rate, stool consistency, and bloody stool score.

The mice were divided into three groups, including the blank control group (control group), the UC model group (DSS group), and the DHP–BBR nanoparticle group (BD group). The administration dose was 225 mg kg^-1^, and it was administered orally for 7 consecutive days.

After 7 days of drug intervention, the mice were fasted for 12 h, and then, blood was collected from the eyeballs of the mice. After blood collection, they were sacrificed by cervical dislocation. The contents of the colon were collected and quickly frozen in liquid nitrogen and stored in a −80°C refrigerator. The colon tissue was collected and photographed. The length of the colon was recorded, and then, the distal colon was transected to take 0.5 cm of the distal colon. The contents were gently scraped off, rinsed in normal saline, and the tissue was placed in the tissue cell fixative solution and left at room temperature for 24 h for use; the remaining part of the colon was also used. After slitting, the contents were removed, rinsed with normal saline, and then, quickly frozen in liquid nitrogen and stored in a −80°C refrigerator for future use. The blood was centrifuged at 4°C under the speed of 3,000 rpm for 15 min and then transferred. The supernatant was used for subsequent processing.

#### 2.7.2 Histology assessment via H&E staining

Hematoxylin and eosin (H&E) staining was used to detect the pathological damage of the colon tissue. The distal colon tissue of the mice in each group was taken, and the contents were removed, rinsed, dried, and then, fixed with 4% paraformaldehyde. We took out the fixed distal colon tissue, rinsed it, and sequentially dehydrated the colon with different concentration gradients of ethanol and then immediately put it in melted paraffin and soaked it in wax. When the wax was soaked in completely, we embedded and cut it into 5 μm thin slices. We stained the slices with hematoxylin, rinsed them until the sections turned blue, and differentiated them until the sections turned red. The blue color was restored after rinsing them with water and dehydrating them after counterstaining with eosin. The slides are sequentially dehydrated with different concentration gradients of ethanol and, finally, treated with xylene-absolute ethanol, xylene solution, etc., and the slides are sealed with a neutral gum. We observed them under a fluorescence microscope (Leica, Wetzlar, Germany) after sealing and selecting different fields of view for each section for the analysis.

#### 2.7.3 Immunohistochemical study of the intestinal barrier in mice

The tissue sections were sequentially placed in a reagent tank for dewaxing and hydration and incubated with 3% hydrogen peroxide for 10 min to eliminate the activity of endogenous peroxidase. We washed it with ultrapure water and phosphate buffered saline, respectively. The sections were repaired by the microwave repair method, blocked with normal goat serum working solution, incubated at room temperature, decanted with the serum, incubated overnight at 4°C with an appropriately diluted primary antibody, and incubated with the HRP-labeled secondary antibody working solution at 37°C for 30 min, DAB solution decans for 3 min, hematoxylin counterstains for 5 min, washed with water, and differentiated with hydrochloric acid alcohol for 20–40 min after returning to blue. It was dehydrated with absolute ethanol and xylene and sealed with neutral resin, and finally, we observed it under a microscope and took pictures to record the experimental results.

### 2.8 M1- and M2-like macrophage transition analysis

RAW 264.7 cells with a good growth status in the logarithmic phase were collected, and the prepared RAW 264.7 cell suspension was seeded in six-well plates after adjusting the density. 3×10^6^ cells/2.5 mL were added to each well, and the cells were cultured at 37°C and 5% CO_2_ for 24 h. When the cells grew 80%–90%, they were treated with LPS (20 μg mL^-1^) for 24 h to polarize M1- and M2-like macrophages. For the shift of M1 toward M2-like macrophages, the M1-like RAW 264.7 cells were treated with 62.5 μg mL^-1^ of BBR, 200 μg mL^-1^ of DHP–BBR, and a blank medium for 24 h. The mRNA expressions of M1- and M2-like maker genes were determined by using the qPCR assay.

The total RNA was extracted from macrophages using TRIzol according to the manufacturer’s instruction. cDNA was transcribed from 1 μg of RNA with a PrimeScript™ RT reagent Kit with gDNA Eraser. Real-time PCR procedures were as follows: 95°C for 10 min, 95°C for 15 s, and 60°C for 60 s. Data were normalized for the actin expression by using the comparative 2^−ΔΔCT^ method. Primers for different genes are listed in Table 1. The gene expression data were examined in duplicates that were repeated three times.

**TABLE 1:** Primer information of the genes in this study.

Name		Primer sequence (5′ to 3′)	Product (bp)
TNF-α	F	ACG​GCA​TGG​ATC​TCA​AAG​AC	116
TNF-α	R	GTG​GGT​GAG​GAG​CAC​GTA​GT	
Arg-1	F	TGC​ATA​TCT​GCC​AAA​GAC​ATC​G	137
Arg-1	R	TCC​ATC​ACC​TTG​CCA​ATC​CC	
ACTIN	F	CCA​TCT​ACG​AGG​GCT​ATG​CT	150
ACTIN	R	CTT​TGA​TGT​CAC​GCA​CGA​TT	

### 2.9 Gut microbiota modulation in mice with ulcerative colitis

We took the fresh feces of mice, mixed the samples of the same group evenly, weighing an appropriate amount, added ethanol, and centrifuged it at room temperature after mixing. We put it in an oven to remove ethanol for later use.

The E.Z.N.A.^®^ Soil DNA kit (Omega Bio-Tek, Norcross, GA, US) was used to extract the total DNA of the microbial community. The method of 1% agarose gel electrophoresis was used to detect the quality of the extracted DNA, and the DNA concentration and purity were determined.

The 16 S rRNA gene V3–V4 variable region was PCR-amplified using 338F (5′-ACT​CCT​ACG​GGA​GGC​AGC​AG-3′) and 806R (5′-GGACTACHVGGGTWTCTAAT-3′). PCR products were recovered using a 2% agarose gel, purified using the AxyPrep DNA Gel Extraction Kit (Axygen Biosciences, Union City, CA, USA), detected by 2% agarose gel electrophoresis, and analyzed with a Quantus™ Fluorometer (Promega, USA) to detect and quantify the recovered product.

The library was constructed using the NEXTFLEX Rapid DNA-Seq Kit (Bioo Scientific Co., Texas, USA) and sequenced using the MiSeq PE300/NovaSeq PE250 platform of Illumina.

### 2.10 Distribution of BD in the colonic tissue

BD loaded with DIL (BD@DIL) was prepared using DIL, BBR, and the DHP. The UC mouse model was established as described previously. The DSS-induced mice were divided into BD@DIL and free-DIL groups, and the normal mice were orally administrated with the same dose of BD@DIL and DIL as the control. Fresh colon tissues were harvested after 4 h and 8 h of administration, respectively. The contents of the tissue were scraped off and washed with PBS, and the colon tissue was embedded in an embedding box using OCT and placed in liquid nitrogen for rapid freezing. Tissue sections were prepared using a frozen sectioning machine (Leica CM1950, Heidelberger, Germany). Cell nuclei were stained using DAPI. The tissue sections were imaged using a fluorescence microscope.

### 2.11 Statistical analyses

Data analyses were performed using the one-way analysis of variance (ANOVA) and nonparametric Tukey’s test. *p* < 0.05 indicated that the difference between the groups was statistically significant.

## 3 Results

### 3.1 Characterization of the DHP

**FIGURE 2:**
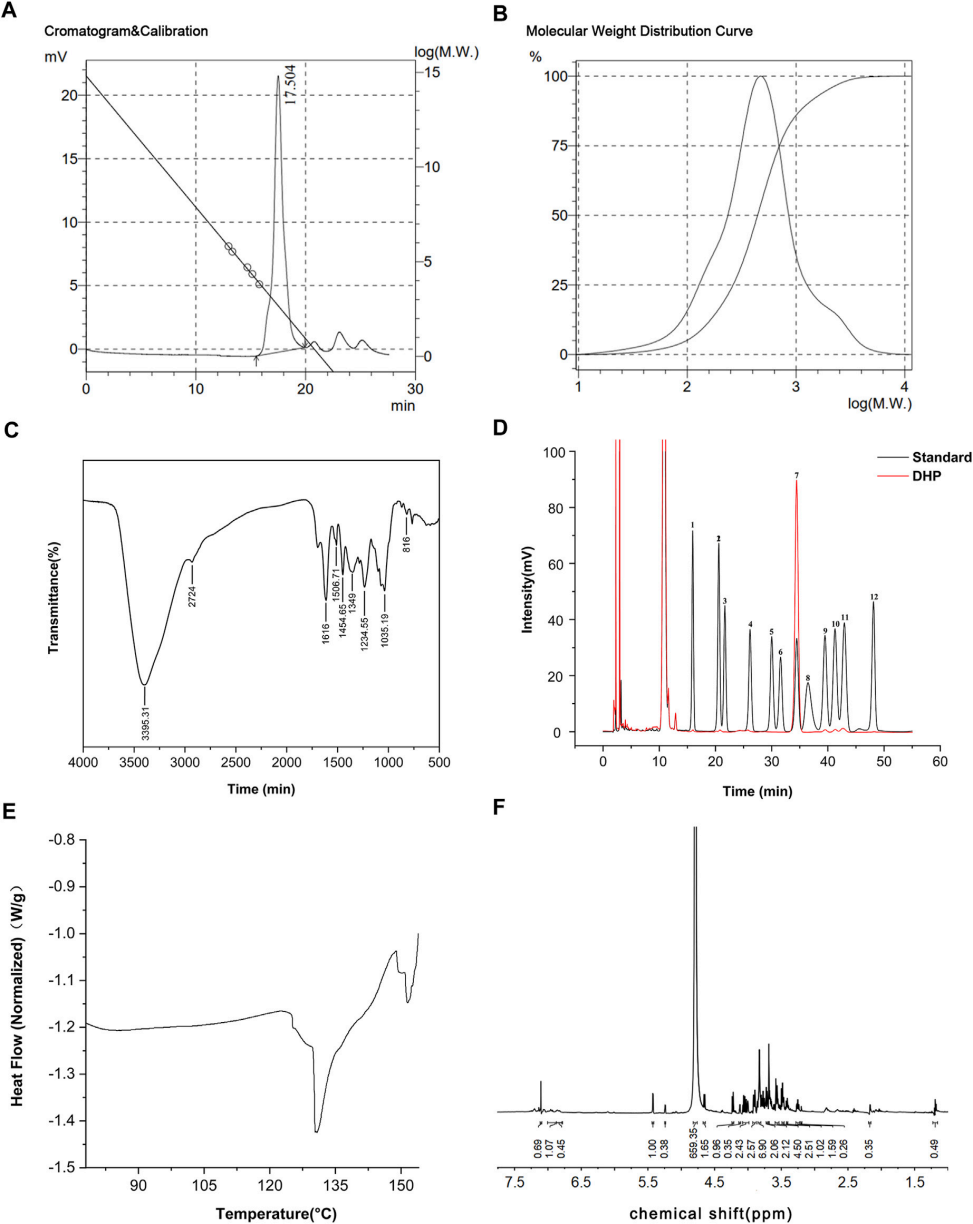
Characterization of the DHP. **(A,B)** Molecular weight standard curve and gel chromatography of the DHP. **(C)** FT-IR spectrum of the DHP. **(D)** HPLC of the DHP. **(E)** DSC of the DHP. **(F)** ^1^H-NMR of the DHP.

The results of the DHP characterization are shown in Figure 2. According to the results of the molecular weight standard curve and gel chromatography, the inter-peak time of the DHP was 15.49 (min) and the average number (Mn) and average molecular weight (Mw) were 280 and 629, respectively (Figure 2A). The molecular weight distribution curve is shown in Figure 2B. The FT-IR spectrum (Figure 2C) of the DHP presented the characteristic absorption bands at 3,395.31 cm^-1^, which suggested that there are multiple hydroxyl groups and indicated the presence of intermolecular or intramolecular O–H bonds. The absorption bands at 2924 cm^-1^ and 1,454.65 cm^-1^ were the stretching vibration peak and bending vibration peak of methylene, respectively. The characteristic absorption bands at 1,616 cm^-1^ were assigned to the carbonyl group. The peak at 1,384–1,062 cm^-1^ indicated the presence of a different form of vibration in the C–O bond of the pyranose ring. The absorption bands at 817 cm^-1^ indicated the presence of an ether structure in the sugar ring. HPLC spectra of the monosaccharide mixed standard were hydrolyzed, which helped in deriving the DHP sample solution, as shown in Figure 2D. Compared with the acetylation results of the monosaccharide mixed standard, the DHP sample is mainly composed of glucose and also contains a small amount of mannose, ribose, glucuronic acid, galacturonic acid, galactose, xylose, arabinose, and fucose. The DSC results (Figure 2E) show that the exothermic peak of the DHP appears at about 130°C, and the endothermic peak occurs at 148°C, that is, a melting occurs. The ^1^H-NMR results (Figure 2F) of the DHP show that δ 5.24 ppm and δ 5.24 ppm belong to the hetero proton signal of α-hexose and the proton signal shift of acetylphthalomethyl at δ 2.17 ppm in the high field region, which further confirms the existence of uronic acid in the polysaccharide structure.

### 3.2 Characterization of BD

**FIGURE 3:**
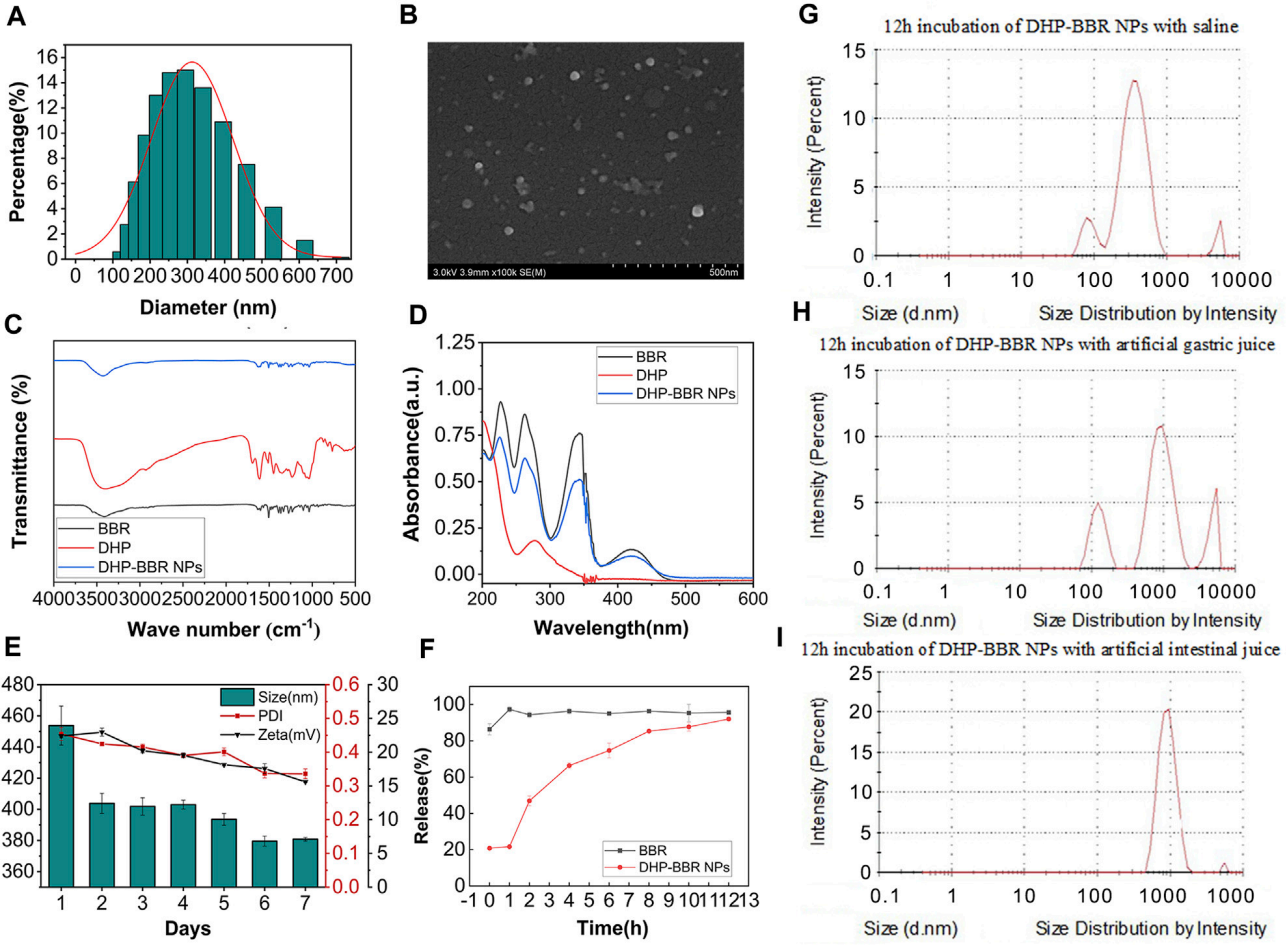
The characterization of BD. **(A)** Size of BD. **(B)** SEM images of BD. **(C)** FT-IR spectrum of BD. **(D)** UV full-wavelength scanning of BD. **(E)** 7-day stability of BD of size, PDI, and zeta potential. **(F)** *In vitro* release of BD and BBR. **(G)** Size change of NPs in saline after 12 h of incubation. **(H)** Size change of NPs in the simulated gastric liquid after 12 h of incubation. **(I)** Size change of NPs in the simulated intestinal liquid after 12 h of incubation. *n* = 3; mean ± SD.

The co-assembly nanoparticles were prepared according to the emulsification and volatilization method. BD was measured immediately after preparation (Figure 3A). The particle size of the BD was about 355 ± 21 nm, the polydispersity index was 0.30 ± 0.06, and the zeta potential was about −10∼16 mV. Lyophilized nanoparticles were measured immediately after re-dissolving them in water. The particle size of the nanoparticles was about 330 ± 30 nm, the PDI was 0.20 ± 0.03, and the zeta potential was −15∼ −20 mV. The zeta potential is very important for the stability of nanoparticles. The larger the absolute value of the potential, the stronger the electrostatic repulsion between particles and the more stable the nanoparticles are. No lyoprotectant was added during lyophilization, but the properties of the resulting nanoparticles were better than those before lyophilization. It is speculated that this is related to the formation mechanism of BD. The DHP and BBR bond with each other through supramolecular forces to form nanoparticles. Under a high-vacuum environment during the lyophilization process, the intermolecular forces are affected by the outside world. The structure of nanoparticles is more compact and stable, and the morphology is more uniform; that is, it shows a smaller particle size and PDI and a higher zeta potential.

The HPLC test showed that BD had a good encapsulation efficiency, which could reach 47% ± 4.1%. Therefore, it is scientific and reasonable to select the DHP as a suitable carrier to encapsulate BBR. The mean size and internal structure of BD were also explored by nanoparticle analysis from SEM data (Figure 3B) and gave a mean size of 300 nm, which is coordinated with the results from the value of 355 nm, as shown in Figure 3A. The SEM image also showed a spherical nanostructure for BD.

From the analysis of FT-IR spectrum data (Figure 3C), it can be seen that 3,405 cm^-1^ is the stretching vibration absorption peak of O–H in sugar molecules and 1,693 cm^-1^ is the characteristic absorption peak of the carboxyl group, which confirms the existence of uronic acid structures in polysaccharides, and in BD, the absorption peak of the carboxyl group is weakened, which confirms that the carboxyl group is involved in the formation of nanoparticles. 1,448 cm^-1^ and 1,350 cm^-1^ are the absorption peaks of C–H bending vibrations.

The full-wavelength scan of BBR in the range of 200–800 nm shows that there are maximum absorption peaks at 227 nm, 263 nm, and 345 nm (Figure 3D). After self-assembly with rhubarb polysaccharides to form BD, the absorbance at the three wavelengths showed a downward trend, which proved that the self-assembled nanoparticles were successfully synthesized by the DHP and BBR.

### 3.3 Stability study of BD

The results of BD storage stability study are as follows. Continuous monitoring of the particle size, PDI, and zeta potential of BD can be obtained from the 7-day monitoring results (Figure 3E); the nanoparticles can be placed continuously at room temperature for several days. During these 7 days, the particle size of the nanoparticles changed relatively after 24 h, and the size changed from 450 nm to 400 nm. Then, after 5 days, it dropped to around 380 nm again. The PDI of nanoparticles remained at around 0.4 for the first 5 days, and on the 6th day, the PDI decreased to nearly 0.30 and remained unchanged after that. The absolute value of the zeta potential of the nanoparticles decreased slightly from about 22 mV in the first 2 days to 15 mV uniformly. On the whole, the stability of the nanoparticles at room temperature is good. With the extension of storage time, the diameter of the nanoparticles tends to drop sharply in a short period of time, but remains stable after long-term storage, and the uniformity of nanoparticles increases with storage. The size changes better over time, but the zeta potential shows a slight decrease. Overall, the nanoparticles have excellent stability at room temperature.

The stability results of BD in different GI fluids are as follows: after co-incubating BD with normal saline for 12 h, the particle size remained unchanged at 322.60 nm and the PDI was 0.35, which was relatively uniform and stable. After co-incubating BD with the simulated gastric fluid for 12 h, the particle size increased to 534.70 nm and the PDI increased to 0.97, indicating that the structure of BD in the simulated gastric fluid was unstable and the formed nanospheres may be partially destroyed. After co-incubating BD with the simulated intestinal fluid for 12 h, the particle size increased to 1,020 nm, which is nearly three times the initial particle size, indicating that the stability of BD in the simulated gastric fluid was better than that in the simulated intestinal fluid (Figures 3G–I). During the process of oral administration, the acid sensitivity of the nanoparticles needs to be further improved to protect the nanoparticles from being destroying in the acid environment of the stomach and being released in large quantities in the large intestine with a higher pH value.

### 3.4 *In vitro* release characteristics

It was verified by *in vitro* release experiments that BD released about 20% of the drug within 1 h in the release medium. The drug stays in the body for 12 h and can basically be released completely (Figure 3F).

### 3.5 Effect of BD on UC-induced mice

#### 3.5.1 Colon length assessment

**FIGURE 4:**
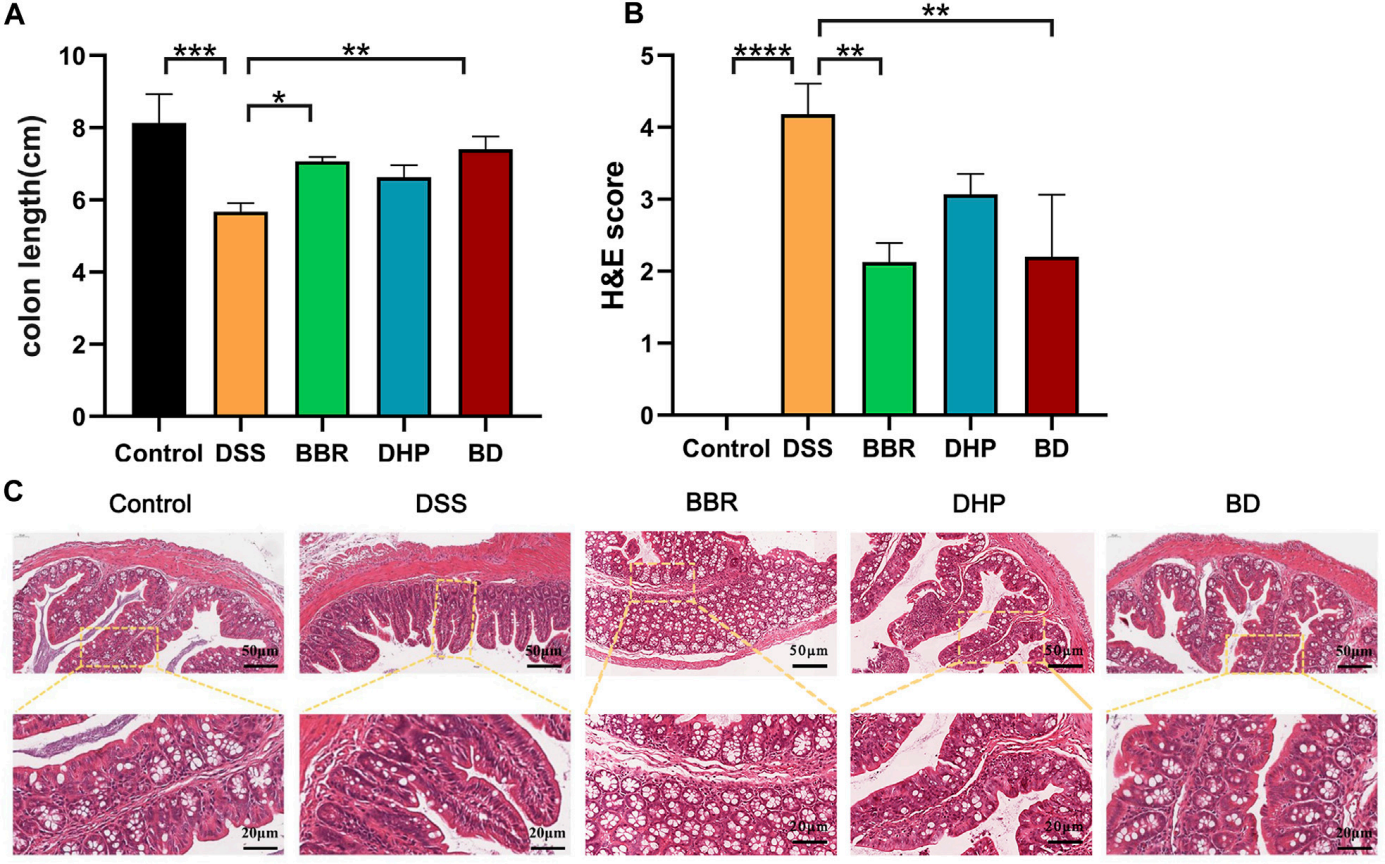
Effect of BD on UC induced mice. **(A)** Colonic length change. **(B)** H&E scores were analyzed by the image J evaluation of H&E staining. **(C)** H&E staining images of every group. (Significance was assessed by using t-test; **p < 0.01, ***p < 0.001, versus Control).

The Disease Activity Index (DAI) assessment indicates the successful modeling of UC mice. In general, the length of the colon in normal mice is about 8 cm, and one of the symptoms of UC induced by DSS is the shortened colon length. The results of this experiment are consistent with that; the length of the colon in the DSS group is about 6 cm, and accompanied by colonic bleeding, BD can increase the colon length of the DSS-induced mice. The colon length of the BD group is maintained at about 8 cm, so BD shows good efficacy in alleviating the shortened colon length of mice with UC (Figure 4A).

#### 3.5.2 Colon histopathological assessment

The histopathological changes in the colon tissue were observed via H&E staining. In the control group, the structure of the colon tissue was intact, the number of goblet cells in the innermost mucosa was more and arranged neatly, and the structure of the crypt in the lamina propria was normal. Compared with the control group, the mucosal structure of the colon in the DSS-induced UC mice was seriously damaged, which was mainly characterized by notable epithelial damage, a decrease in the number of goblet cells, disappearance of the crypt, and severe edema in the muscle layer. However, compared with the DSS group, the colon injury of the BD group treated with BD was significantly improved, mainly in the effective recovery of the crypt structure and improving the degree of the muscular edema. Therefore, BD can effectively alleviate the injury of the colon tissue in mice with UC (Figures 4B, C).

#### 3.5.3 Changes of the colon barrier function

**FIGURE 5:**
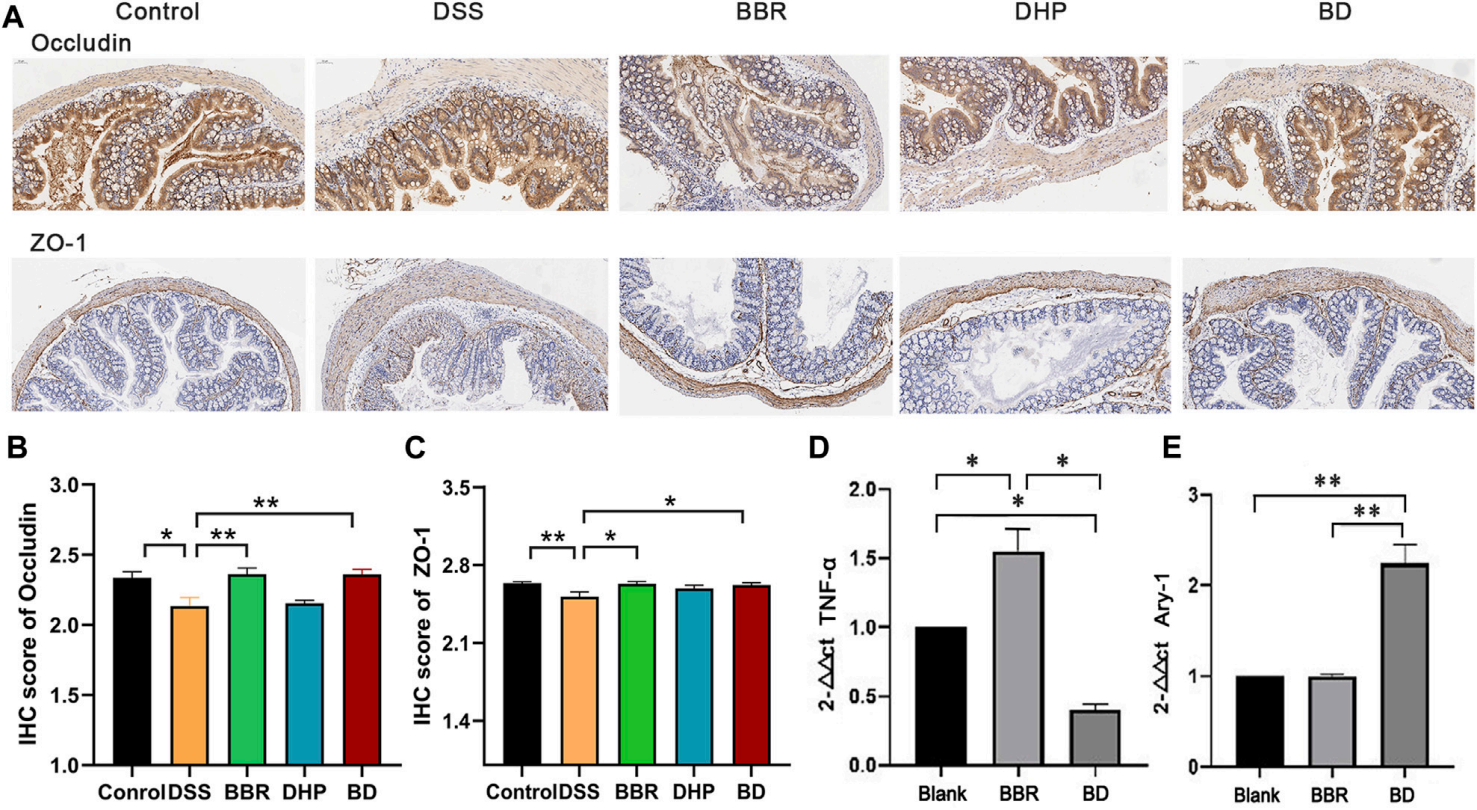
Biochemical index of in UC mice after oral administration of different groups. **(A)** Immunohistochemical staining of ZO-1 and occludin of colon in normal mice (Control), DSS-induced UC mice after the treatment of DI water (DSS) and BD. **(B)** IHC score of occludin by the image J evaluation. **(C)** IHC score of ZO-1 by the image J evaluation. **(D)** The relative level of TNF-α and **(E)** Arg-1 of RAW 264.7 cells incubated with LPS after the treatment of blank media (Blank), BBR and BD. Significance was assessed by using t-test. *p < 0.05, **p < 0.01, versus Control or Blank.

The intestinal tight junction protein is a physical barrier located at the top between colon epithelial cells and endothelium, allowing ions and small molecules to selectively pass between cells. The expression of the tight junction protein can affect the state of mucosal inflammation by regulating mucosal homeostasis and intestinal permeability. As a classic model of UC, DSS intervention may destroy the intestinal epithelial structure through its own cytotoxicity, promoting tight junction protein translocation in the colon mucosa and flora infiltrating the colon tissue, which induces inflammation. In the blank control group, the expression levels of transmembrane proteins, occludin and zonula occludens-1, were relatively high. In the DSS group, the expression of both proteins decreased and the downward trend of ZO-1 was more evident. After the intervention of BD treatment, the expression of the two proteins in the colon of the mice returned to the level of the control group (Figure 5A). The results of the immunohistochemistry (IHC) score are shown in Figures 5B, C.

#### 3.5.4 BD can transit M1- to M2-like macrophages

To investigate the effect of LPS on RAW 264.7 cells, the morphological characteristics of RAW 264.7 cells before and after LPS (20 μg mL^-1^) treatment were observed using an optical microscope. Before LPS treatment, RAW 264.7 cells appeared oval and undifferentiated. The morphology of the cells changed significantly after LPS of 20 μg mL^-1^ incubation for 24 h. Therefore, LPS treatment caused significant polarization of M1- and M2-like macrophages.

Compared with the blank group, the M1-type marker TNF-α content in the BD group was decreased to different degrees, while the mRNA expression level of the M2 marker Arg-1 in the DHP–BBR group was significantly increased after administration for 24 h (Figures 5D, E).

### 3.6 Effect of BD on the gut microbiota in UC mice

#### 3.6.1 Beta diversity of the gut microbiota in mice

**FIGURE 6:**
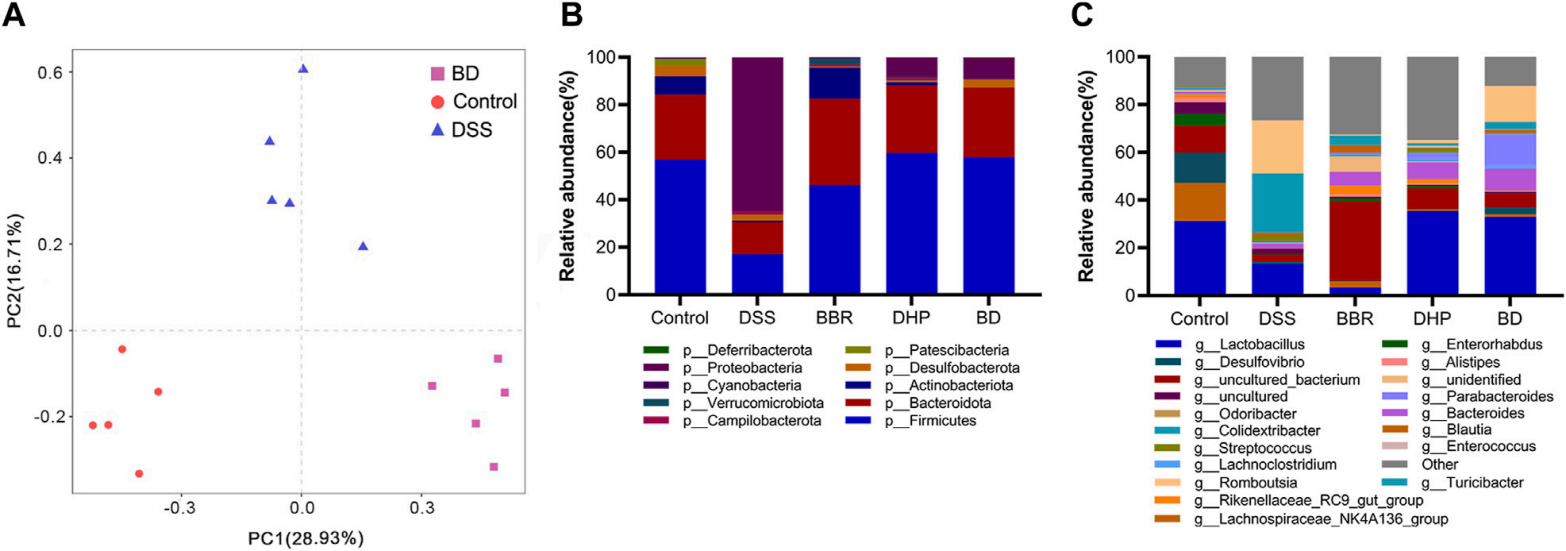
Gut microbiota analysis after oral administration in different groups. **(A)** Principal components analysis (*n* = 5). **(B)** Relative abundance of the gut microbiota of mice in each group at the phylum level. **(C)** Relative abundance of the gut microbiota of mice in each group at the genus level.

Beta diversity analysis was further performed on BD, control, and DSS groups. The results showed that there was a significant difference between these three groups, confirming their significant differences in flora composition. The intervention of BD could effectively repair gut microbiota disorders in UC mice (Figure 6A).

#### 3.6.2 BD administration altered the relative abundance of taxa at multiple levels in UC mice

Next, we focused on the composition of the phylum level of five groups of mice gates. From the relative abundance levels of *Firmicutes* in BBR and DHP groups, the DHP promoted its recovery more. Thus, the intervention of BD fully exploited the advantages of BBR and the DHP. Among the other dominant phylum, the relative abundance level of *Bacteroidetes* was significantly lower in the DSS group, which increased in the BBR group and returned to normal levels in DHP and BD groups compared with the control group. This shows that the DHP in BD plays a role in regulating the structure of the bacterial population. (Figure 6B).

The composition of the mice in each group at the genus level was analyzed. The results showed that the relative abundance of *Lactobacillus* was significantly lower in DSS and BBR groups compared to the control group, but DHP and BD groups had similar levels to the control group, this echoed the changes in the microbiota at the phylum level. Based on the aforementioned results, it was speculated that the effect of BD on the structure of the gut microbiota was mainly due to the DHP (Figure 6C).

### 3.7 BD@DIL NP distribution in the colonic tissue

**FIGURE 7:**
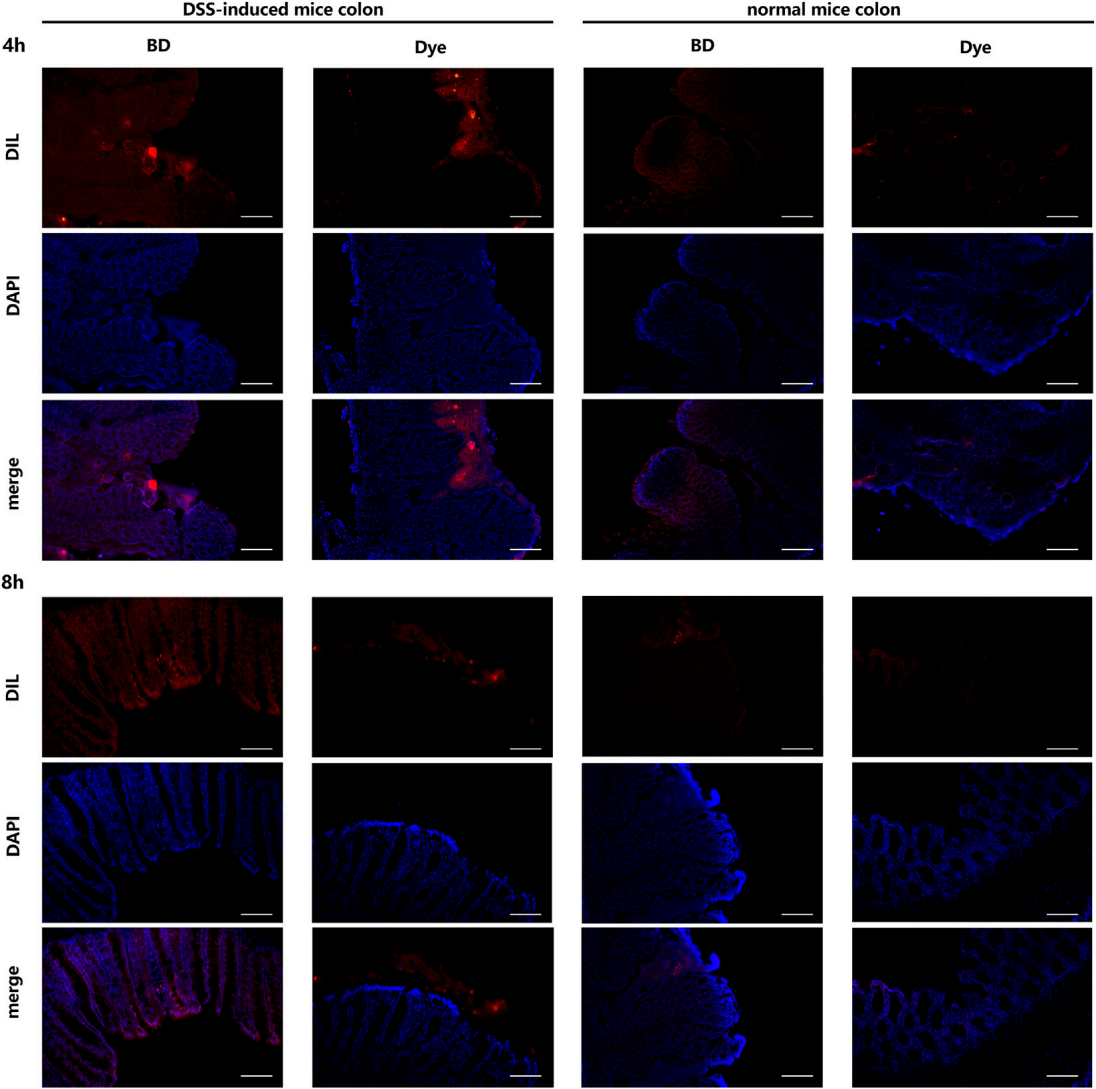
Distribution of BD@DIL NPs in the colonic tissue after 4 h and 8 h of administration. Scale bar = 200 μm.

The drug delivery effect of BD@DIL was observed via microscopy, and the results of the DSS-induced mice at 4 h and 8 h showed that the red fluorescence of the BD group was clearly distributed in the colonic tissue, while the results of the DIL group showed that DIL without NPs failed to penetrate the tissue. It was confirmed that oral administration of BD resulted in the delivery of BBR to the colon and its absorption by colonic cells and showed more retention in the colon compared to free DIL (Figure 7).

## 4 Discussion

The mucin-type glycosylation process is a specific and complex post-translational modification of proteins. According to studies, the synthesis of mucin-type O-glycans has been proved to be closely related to it (Da Silva et al., 2014). It is particularly important for the structural stability of mucins in the gastrointestinal tract. A deficiency in O-glycosylation can lead to impaired mucin expression and the destruction of the mucosal barrier, leading to the microbial activation of inflammatory bodies, such as caspase-1, interleukin (IL)-1β, and IL18, which in turn promotes inflammation and leads to severe spontaneous bacterial-dependent colitis (Larsson et al., 2011; Bergstrom et al., 2016). O-glycans is the main form of glycosylation, which is characterized by the fact that oligosaccharides are linked to hydroxyl groups of serine or threonine residues to form O-linked glycoproteins under the action of serine or threonine residues of the polypeptide chain (Timpte et al., 1988; Gupta and Jentoft, 1989). The O-linked N-acetylgalactosamine (O-GalNAc) sugar is especially related to the mucous membrane of the respiratory, genitourinary, and gastrointestinal tract (Corfield and Berry, 2015; Martens et al., 2018). Therefore, it is inferred from the results of HPLC that a small amount of mannose, xylose, GalNAc, and other components in Rheum polysaccharides can achieve therapeutic effects by promoting the glycosylation process and reducing the inflammatory response.

In tumor microenvironment-targeted drug delivery systems, strategies are often employed to enhance the penetration and retention effect (EPR effect), which is also associated with the colonic inflamed tissue. Between the colonic mucosa and the intestinal lumen, there is a grid-like space connected by mucin (mainly, mucin-2), which is the mucus layer. Crucially, it is the only barrier that isolates the colon tissue from a large number of microorganisms colonizing the colonic lumen. When local inflammation occurs, the mucus layer is degraded, the grid-like structure is destroyed, and gaps in the mucus layer become larger, which were originally isolated in large quantities (Wu et al., 2022). The nanoparticles (less than 500 nm) in the outermost layer of the mucus layer can penetrate the colon tissue and are easily absorbed by intestinal epithelial cells and immune cells of the diseased colon tissue, while the larger-diameter drug particles are still sequestered in the intestinal lumen (Zhang et al., 2021). The diameter of BD is 330 ± 30 nm, which can achieve passive aggregation to the site of inflammation for achieving better drug efficacy.

The intestinal epithelial mucus is adhered by bacteria such as *Lactobacilli*, *Bifidobacteria*, and *Streptococci*, which play a regulatory and protective role in the intestinal mucosal barrier (Grander et al., 2018). ZO-1 and occludin are two tight junction proteins that cannot be ignored and have functions such as repairing intestinal epithelial damage and maintaining cell polarity (Ishikawa et al., 2017; Hale, 2020). Increasing evidence suggests that the intestinal microbiota is involved in signaling in the intestinal epithelium and can influence the intestinal barrier function by regulating the distribution and expression of tight junction proteins (Roy and Trinchieri, 2017; Riehl et al., 2019). *In vivo* efficacy experiments showed that BD could significantly inhibit the shortening of the colon length in mice with UC, effectively restore the crypt structure and mucosal thickness of the colon tissue in mice with UC, and upregulate the expression of the intestinal tight junction protein, occludin. The expression causes the effective repair of the intestinal barrier integrity. In conclusion, after preliminary exploration, BD can effectively relieve the symptoms of the DSS-induced UC mice.

With the development and popularization of next-generation gene-sequencing technology, the relationship between the intestinal flora and human health and disease is gradually becoming clear and the “black box” of the intestinal flora is gradually being opened. The intestinal flora is mainly found in the colonic segment of the large intestine, where a number of viable bacteria reach 1,012–1,014. The intestinal microbiota has physiological functions related to nutrition, immune system, and host defense. Many studies have been conducted to demonstrate the altered composition and function of the gut microbiota in IBD patients, which is closely related to the pathogenesis of IBD. For instance, the concentration of SCFAs has been reported to decrease in IBD patients, as a result of butyrate-producing bacteria, such as *fusobacterium prausnitzii* and *Clostridium clusters IV*, *XIVa*, and *XVIII* (Takahashi et al., 2016). The decreased production of SCFAs affects the differentiation and expansion of Treg cells and the growth of epithelial cells (Atarashi et al., 2013), which plays an important role in maintaining the intestinal homeostasis. The short-chain fatty acid-producing bacterium *Clostridium* perfringens cluster induces the differentiation and amplification of colonic Tregs by producing butyric acid in healthy organisms. The relative abundance of *Clostridium* in patients with inflammatory bowel disease is statistically lower than that in normal humans, and its reduced amount is positively correlated with the recurrence chances of Crohn’s disease. On the other hand, the number of sulfate-reducing bacteria, such as *Desulfovibrio*, is higher in IBD patients, resulting in the production of hydrogen-sulfate that damages intestinal epithelial cells and induces mucosal inflammation (Rowan et al., 2010; Nishida et al., 2018). Collectively, these findings suggest that alterations in the gut microbiota are closely related to the pathogenesis of IBD.

The results of the phylum level analysis showed that *Firmicutes, Bacteroidetes*, and *Proteobacteria* were the three most dominant phyla in all the samples. It was demonstrated that the proportion of *Firmicutes* was negatively correlated with IBD (Quévrain et al., 2016; Wu et al., 2020). There was a significant increase in *Proteobacteria* and a significant decrease in *Firmicutes* in the DSS group, but the control group levels were restored with the intervention of BD. The analysis of the genus level results revealed that BD had a significant enrichment effect on *Lactobacillus*. The members of the genus *Lactobacillus* that belong to the phyla *Firmicutes* are the most important probiotic bacteria of the gut microbiome. *Lactobacilli* in the intestine associated with the epithelium repair the gut barrier, enhance barrier defense, and improve the host immune response (Martín et al., 2019). In addition, *Lactobacillus* species exhibit microbial roles by competitively excluding opportunistic pathogens from inhabiting functional niches in the gut, restraining the attachment of pathogens on the epithelium, and directly killing pathogens by producing lactic acid, acetic acid, propionic acid, bacteriocins, and reactive oxygen species (ROS) (Cristofori et al., 2021; Dempsey and Corr, 2022).

In addition, studies have shown that berberine is useful in the treatment of type II diabetes, inflammatory colitis, depression, and other neurological disorders. Berberine significantly increased the community richness and diversity of the intestinal flora. Qi-Kui Chen et al. (Zhang et al., 2019) studied the effects of berberine intervention on various indicators in db/db mice and showed that berberine reduced the food intake, body weight, blood glucose, and HbA1c levels, effectively restoring the short-chain fatty acid content, reducing serum LPS levels, alleviating intestinal inflammation, and repairing the intestinal barrier structure in mice (Habtemariam, 2020). Berberine intervention altered the intestinal flora of mice, increasing the relative abundance of short-chain fatty acid-producing bacteria such as *Butyricimonas*, *Coprococcus*, and *Ruminococcus*, while reducing the number of conditionally pathogenic bacteria such as *Prevotella* and *Proteus*. Thus, berberine could alleviate intestinal inflammation by modulating the composition of the intestinal flora.

In addition to its significant anti-ulcerative colitis efficacy, BD also have many unique advantages, such as the self-assembly of only two natural components of the DHP and BBR, without any toxic side effects in the body; there is no drawback that shows us that drug additives may pose any potential harm to the body. It is also recorded in traditional Chinese medicine ancient books that the combination of *Rheum palmatum* L*.* and *Coptis chinensis* Franch*.* is used to treat gastrointestinal diseases. At present, the mechanism for the compatibility of *Rheum palmatum* L*.* and *Coptis chinensis* Franch*.* is mostly explained by the theory of the pairing of rhein and BBR; there is no research on the combination of the DHP and BBR. This study provided a reference for the modern scientific use of the traditional Chinese medicine-based polysaccharides and nanotechnology strategies.

## 5 Conclusion

This study characterized the properties of the DHP for further fabrication of the nanocomposites together with BBR. Then, BD was prepared by the co-assembling strategy and the particle size, morphology, UV–Vis and FT-IR characteristics, encapsulation efficiency, and stability of the nanoparticles were investigated, respectively. The DSS-induced C57BL/6 UC mouse model was established, and the remission of colitis symptoms and the recovery of the intestinal barrier function in mice were determined by measuring the length of the mouse colon and H&E and immunohistochemistry staining. The results showed that the DHP and BBR were co-assembled to form nanoparticles, and BD can effectively relieve the symptoms of the UC mouse induced by DSS by regulating the gut microbiota and repairing the gut barrier integrity because BD shows longer retention on the colon tissue and reacts with the microbiota and mucus thoroughly. Interestingly, BD can enrich more probiotics than free BBR and DHPs. This paper depicted the compatibility mechanism of *Rheum palmatum* L*.* and *Coptis chinensis* Franch*.* based on the theory of the pairing of rhubarb polysaccharides and BBR, which provided a reference for the modern scientific use of the traditional Chinese medicine pairing of polysaccharides based on co-assembled nanotechnology strategies.

## Data Availability

The datasets presented in this study can be found in online repositories. The names of the repository/repositories and accession number(s) can be found below: https://www.ncbi.nlm.nih.gov/sra/PRJNA982533.
